# Comparison of surgery versus observation for small angle intermittent exotropia

**DOI:** 10.1038/s41598-020-61568-0

**Published:** 2020-03-13

**Authors:** Jae Yun Sung, Hee Kyung Yang, Jeong-Min Hwang

**Affiliations:** 1 Department of Ophthalmology, Chungnam National University College of Medicine, Chungnam National University Hospital, Daejeon, Korea; 2 Department of Ophthalmology, Seoul National University College of Medicine, Seoul National University Bundang Hospital, Seongnam, Korea

**Keywords:** Eye abnormalities, Ocular motility disorders

## Abstract

In young children with small angle exotropia, making decisions for the individual patient whether to perform surgery or not, and choosing the optimal time for surgical intervention are quite difficult. We aimed to compare the long-term outcomes of small angle intermittent exotropia of 20 prism diopters (PD) or less after observation versus strabismus surgery. A retrospective study was performed on 164 patients aged 3 to 13 who underwent surgical intervention or observation with or without conservative management for intermittent exotropia of 14 to 20 PD. The minimum follow-up period was 2 years. The average follow-up period was 3.9 ± 2.2 years in the observation group and 4.5 ± 2.3 years in the surgery group. At the final examination, the mean angle of deviation at distance was 11.1 ± 8.9 PD in the observation group and 9.0 ± 7.5 PD in the surgery group, which was not significantly different (*P* = 0.121). Changes in sensory outcome and fusional control were not significantly different between both groups (*P* = 0.748 and *P* = 0.968). Subgroup analysis including patients with poor fusional control also showed similar results. By multivariate analysis, the type of surgery, unilateral recess-resect procedure, was the only predictive factor of good motor outcome in the surgery group. In conclusion, long-term surgical outcomes in small angle exotropia did not appear to be more satisfying than observation in terms of motor and sensory outcomes.

## Introduction

Intermittent exotropia is the most common form of exodeviation in childhood^1,2^, and often requires surgical intervention. Surgery is considered when there is a progressively increasing angle of deviation, increasing frequency of deviation, deterioration in binocular vision or cosmetic problems^3,4^. The basic goal of surgery is to restore ocular alignment and to preserve binocular function. Although many studies have reported treatment strategies for intermittent exotropia, clear guidelines for clinicians are lacking. Particularly in young children with small angle exotropia, making decisions for the individual patient whether to perform surgery or not, and to choose the optimal time for surgical intervention is quite difficult.

The natural history of intermittent exotropia remains unclear due to the lack of prospective studies. There have been few retrospective studies^5–9^ of intermittent exotropia with variable ranges of exodeviation (10 to 40 prism diopters, PD). Lee *et al*.^10^ investigated the clinical course of patients with small angle exotropia of 10 to 18 PD. After 2 years of follow-up, nearly half of patients reached 20 PD or underwent surgery and a constant deviation at distance on the initial examination was associated with a higher probability of surgical intervention^10^. However, the final outcomes of patients who were managed conservatively or underwent surgery were not provided. Buck *et al*.^11^ performed an observational cohort study on 371 children with previously untreated exotropia under 12 years of age regarding outcomes of surgery versus non-surgical treatment including spectacle lens, occlusion, exercise and prisms. They concluded that surgery was the only intervention associated with significant improvements in the angle of deviation and fusional control. However the range of exodeviation was wide in both groups (10 to 60 PD), and outcomes for surgery were assessed only after 6 months which is too short considering the continuous exodrift occurring years after exotropia surgery^12–18^.

To the best of our knowledge, there is no report that directly compared the natural course of observation versus surgery in patients with small angle exotropia of ≤20 PD. The purpose of this study was to investigate whether surgery is useful for the treatment of small angle exotropia. We compared the long-term outcomes of small angle exotropia in the observation group and in the surgery group, and determined predictive factors affecting motor outcomes in each group.

## Results

### Baseline characteristics

Among 164 patients included in this study, 82 patients in the observation group and 82 patients in the surgery group, the baseline characteristics were not significantly different between the two groups except for fusional control. Eighteen percent of patients in the observation group and 51% of patients in the surgery group showed poor fusional control (*P* < 0.001, Chi-square test). The mean angle of deviation at distance was 17.9 ± 1.8 PD in both groups (*P* = 1.000, Student’s t-test). The mean angle of deviation at near was 16.5 ± 3.4 PD in observation group and 16.0 ± 3.1 PD in surgery group (*P* = 0.336, Student’s t-test) (Table 1).

**Table 1:** Baseline characteristics.

	Observation (n = 82)	Surgery (n = 82)	*P*-value
Male gender	38 (46.3%)	45 (54.9%)	0.274^†^
Age at onset (y)	4.9 ± 2.5 (3–11)	4.2 ± 2.5 (3–10)	0.118^‡^
Age at diagnosis/surgery (y)	6.1 ± 3.1 (3–13)	6.3 ± 2.6 (3–13)	0.563^‡^
Refractive errors (D)	−0.9 ± 2.3	−0.5 ± 2.2	0.210^‡^
**Initial/preoperative deviation**			
Distance (PD)	17.9 ± 1.8 (14–20)	17.9 ± 1.8 (14–20)	1.000^‡^
Near (PD)	16.5 ± 3.4 (10–20)	16.0 ± 3.1 (10–20)	0.336^‡^
Anisometropia	12 (14.6%)	8 (9.8%)	0.340^†^
Amblyopia	8 (11.1%)	3 (3.9%)	0.097^†^
**Associated features**			
DVD	0	0	
A or V pattern	0	0	
Lateral incomitancy	0	0	
Vertical deviation> 5PD	6 (7.3%)	3 (3.7%)	0.304^§^
Inferior oblique overaction	13 (15.9%)	12 (14.6%)	0.828^†^
Superior oblique overaction	4 (4.9%)	2 (2.4%)	0.405^c^
Fixation preference	23 (28.0%)	32 (39.0%)	0.137^†^
**Poor fusional control**			
Distance	15 (18.3%)	42 (51.2%)	**<0.001**^†^
Near	8 (9.8%)	12 (14.6%)	0.340^§^
Good stereopsis	44/68 (64.7%)	44/74 (59.5%)	0.520^†^
Follow-up period (y)	3.9 ± 2.2 (2.0–12.2)	4.5 ± 2.3 (2.0–10.0)	0.131^‡^
**Type of surgery**			
RR		43 (52.4%)	
ULR		34 (41.5%)	
BLR		5 (6.1%)	

y = year(s); D = diopters; PD = prism diopters; DVD = dissociated vertical deviation; RR = lateral rectus muscle recession and medial rectus muscle resection; ULR = unilateral lateral rectus recession; BLR = bilateral lateral rectus recession; Significant P values are expressed in bold characters.

^†^Chi-square test.

^‡^Student’s t-test.

^§^Fisher’s exact test.

### Motor outcomes

**Figure 1:**
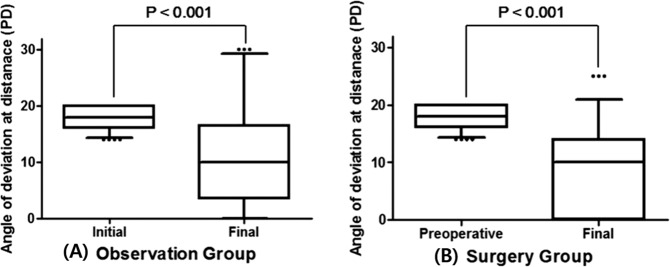
Angle of exodeviation at distance in the initial and final examinations of the Observation group (**A**) and Surgery group (**B**). In both groups, the final angle of exodeviation was significantly smaller than the initial angle of exodeviation (*P* < 0.001 by Student’s t-test). The amount of change was not significantly different between the two groups (−6.8 PD vs −8.8 PD, *P* = 0.136, *P*aired t-test). PD = prism diopters.

The mean follow-up duration was 3.9 ± 2.2 years (range, 2.0 to 12.2 years) in the observation group and 4.5 ± 2.3 years (range, 2.0 to 10.0 years) in the surgery group (*P* = 0.131). In terms of estimating the final angle of exodeviation, 5 patients with consecutive esotropia in the surgery group were excluded from the analysis as it may underestimate the average angle of exodeviation. At the final follow-up examination, the mean angle of exodeviation at distance decreased to 11.1 ± 8.9 PD in the observation group and 9.0 ± 7.5 PD in the surgery group (*P* = 0.121). In both groups, the final exodeviation at distance had significantly reduced compared to the initial/preoperative deviation (*P* < 0.001, Student’s t-test) **(**Fig. 1**)**.

The mean angle of exodeviation at near also decreased to 10.8 ± 9.9 PD in the observation group and 10.0 ± 8.3 PD in the surgery group at the final examination, which was not significantly different between both groups (*P* = 0.561, Student’s t-test). At the final follow-up examination, 36 (44%) patients in the observation group and 37 (46%) patients in the surgery group had good motor outcomes, which was not significantly different between both groups (*P* = 0.754, Chi-square test).

Among those who received surgery, 39 patients (48%) had undercorrection and 5 (6%) had overcorrection. Overall, 37 patients (45%) required reoperation for recurrent exotropia after a mean duration of 3.3 ± 1.7 years (range, 1.0 to 7.5 years) from the first surgery. The five patients (6%) with consecutive esotropia had an esodeviation of 8 to 30 PD (Table 2).

**Table 2:** Outcomes in the observation group and surgery group.

	All patients (n = 164)			Patients with poor fusional control (n = 57)		
	Observation (n = 82)	Surgery (n = 82)	P value	Observation (n = 15)	Surgery (n = 42)	*P*-value
**Final deviation**						
ET < 5PD & XT < 10PD	36 (43.9%)	38 (46.3%)	0.754^†^	8 (53.3%)	19 (45.2%)	0.590^‡^
XT ≥ 10PD	46 (56.1%)	39 (47.6%)		7 (46.7%)	21 (50.0%)	
ET ≥ 5PD	0 (0.0%)	5 (6.1%)		0 (0.0%)	2 (4.8%)	
**Fusional control**^**§**^						
Improved	44 (53.7%)	44 (53.7%)	0.968^†^	14 (93.3%)	23 (54.8%)	**0.007**^‡^
Stationary	27 (32.9%)	28 (34.1%)		1 (6.7%)	19 (45.2%)	
Decreased	11 (13.4%)	10 (12.2%)				
**Stereopsis**^**¶**^						
Improved	28 (41.2%)	29 (39.2%)	0.748^†^	5 (35.7%)	15 (41.7%)	0.780^†^
Stationary	32 (47.1%)	33 (44.6%)		5 (35.7%)	14 (38.9%)	
Decreased	8 (11.8%)	12 (16.2%)		4 (28.3%)	7 (19.4%)	

ET = esotropia; PD = prism diopters; XT = exotropia; Significant P values are expressed in bold characters.

^†^Chi-square test.

^‡^Fisher’s exact test.

^**§**^Improved/Decreased fusional control was defined as a change of one grade or more at the final follow-up examination.

^**¶**^Improved/Decreased stereopsis was defined as a change of 2 or more octaves at the final follow-up examination.

### Stereopsis

Sixty-eight patients in the observation group and 72 in the surgical group were able to test stereoacuity. Good stereopsis was present in 65% in the observation group and 60% in the surgical group at the final follow-up examination (*P* = 0.520, Chi-square test). In the observation group, improved stereopsis was found in 41% and decreased stereopsis in 12% of patients. In the surgery group, improved stereopsis was observed in 39% and decreased stereopsis in 16% of patients. The sensory outcome was not significantly different between both groups (*P* = 0.748, Chi-square test) (Table 2).

### Fusional control

Forty-four (54%) patients showed improvement in fusional control at distance in both groups. Decreased fusional control at distance was found in 13% in the observation group and 12% in the surgery group. The change in fusional control at near and distance were not significantly different between both groups at the final follow-up examination (*P* = 0.135 and *P* = 0.968, Chi-square test) (Table 2).

## Myopic Shift

From the initial to final visit, refractive errors showed a myopic shift in both groups. There were no significant differences in the amount of myopic shift between the observation group and surgery group (−0.34 D/year vs −0.42 D/year; *P* = 0.130, Student’s t-test). Fast myopia progression rates were not significantly different between two groups (28% vs 34%; *P* = 0.399).

### Subgroup analysis in patients with poor fusional control

Fifteen (18%) patients in the observation group and 42 patients (51%) in the surgery group presented with poor fusional control on initial/preoperative examination. The baseline characteristics including gender, age at diagnosis/surgery, initial stereopsis and initial/preoperative angle of deviation at near and distance were not significantly different between the two groups (*P* > 0.05 by Fisher’s exact test and Mann-Whitney U test). The mean angle of exodeviation at distance was 17.5 ± 1.6 PD in the observation group and 18.0 ± 1.6 PD in the surgery group (*P* = 0.308, Mann-Whitney U test). In the surgery group, 43% of patients underwent ULR, 7% underwent BLR and 50% underwent unilateral recession-resection (RR).

At the final follow-up examination, the rate of improvement in fusional control at distance was significantly greater in the observation group compared to the surgery group (93.3% vs 54.8%, *P* = 0.007, Fisher’s exact test) (Table 2). There were no significant differences in the final angle of deviation at distance (9.9 ± 10.4 PD vs 9.3 ± 8.0 PD, *P* = 0.992 by Mann-Whitney test) and the rate of improvement in stereopsis (35.7% vs 41.7%, *P* = 0.780, Chi-square test) between the observation group and the surgery group with poor fusional control.

### Predictive factors for good/poor outcome

In the observation group, inferior oblique overaction was the only predictive factor associated with poor motor outcome by univariate analysis (*P* = 0.032) but not by multivariate analysis.

In the surgery group, older age at surgery, initial good stereopsis and RR procedure were predictive factors associated with good motor outcome in univariate analysis (*P* = 0.007, *P* = 0.044 and *P* = 0.005), whereas only RR procedure was significantly associated with good motor outcome by multivariate analysis (*P* = 0.005, multivariate logistic regression). Gender, initial/preoperative angle of exodeviation at distance and near, refractive errors, initial fusional control, anisometropia, amblyopia, vertical deviation, and fixation preference were not significantly associated with motor outcomes.

## Discussion

Our study provides important information that can help clinicians make treatment decisions in patients with small angle exotropia of 14 to 20 PD. After a mean follow-up of 4.2 years, approximately half of patients had good motor outcomes in both the observation group and surgery group. Long-term surgical outcomes in small angle exotropia did not appear to be more satisfying than conservative management in terms of motor and sensory outcomes. In particular, patients who presented with poor fusional control also showed similar results by subgroup analysis. Meanwhile, the type of surgery, RR procedure, was the only significant factor associated with good surgical outcome in small angle exotropia.

The natural history of intermittent exotropia remains unclear. It has been generally thought that the deviation gradually increases and decompensates to a constant deviation^9,19,20^. von Noorden^9^ reported that 75% of untreated patients (age 5 to 10 years) showed progression over an average follow-up period of 3.5 years. However, in some cases, the exodeviation remains stable for many years, and may even spontaneously improve. Hiles *et al*.^5^ found that exodeviation at distance decreased by 5 PD (23 to 18 PD) in 48 unoperated intermittent exotropia patients after a mean follow-up of 11.7 years. Their study included patients with an initial exodeviation of 18 to 40 PD and 81% of patients received non-surgical therapy. Rutstein and Corliss^6^ enrolled exotropia patients regardless of their size of deviation including deviations even greater than 50 PD. Among them, 80% of patients had received non-surgical therapy and the exodeviation at distance decreased by 3 PD (17 to 14 PD) after a mean follow-up of 10 years. In our study, the mean exodeviation at distance decreased by 6.8 PD (from 17.9 to 11.1 PD) in the observation group after 3.9 years of follow-up. Our findings show a greater decrease in exodeviation despite the relatively shorter follow-up duration compared to previous studies. This might be due to the difference in the initial angle of exodeviation or the proportion of patients who received non-surgical treatment. In our study, the initial angle of exodeviation was smaller (14 to 20 PD). Nearly all patients (91%) in the observation group received non-surgical treatment such as part time occlusion and/or over-minus lens, and the majority of patients showed good compliance to treatment which may affect outcomes.

In the initial/preoperative examination, there was no significant difference in the good stereopsis rates between both groups although the distance fusional control was significantly worse in the surgery group. This can be explained by the good fusional control during near fixation in both groups. Near fusional control was relatively good in the surgery group even when distance fusional control was poor. Similar findings have been observed in previous studies of intermittent exotropia, in which near stereoacuity was protected by stable control during near fixation and the intermittent nature of the deviation^21,22^.

One interesting point is that the change in the angle of deviation showed no difference between the observation group and the surgery group in our study, as nearly half of the patients who received surgery showed undercorrection and recurrence of exotropia upto 25 PD. Further analysis was performed to evaluate the possible difference of outcome between two groups in terms of progressively increasing angle of deviation. Progressively increasing angle of deviation was defined as an increase in exodeviation of 5 PD or more^10^. Eight patients (9.8%) in the observation group and 3 patients (3.7%) in the surgery group showed progressively increasing angle of deviation after a mean follow-up of 3.9 years and 4.5 years, which was not significantly different (*P* = 0.210). There was no significant difference in motor and sensory outcomes between the observation group and the surgery group. Subgroup analysis in patients with poor fusional control was performed to exclude the possibility of selection bias of more patients with poor fusional control in the surgery group. The motor and sensory outcomes in the surgery group were not superior to those of the observation group even in patients with poor fusional control at distance. Considering that RR procedure was associated with good motor outcome, we compared the motor outcome of patients who underwent RR with the observation group, but there was no significant difference between the two groups (58.1% vs 43.9%; *P* = 0.130). Sensory outcomes showed similar results (*P* = 0.797).

The surgical success rates of small to moderate angle exotropia (15 to 35 PD) have been reported to be 56.1% to 78%^23–27^. In our study, 46% of patients showed successful outcome after a mean follow-up of 4.5 years. The relatively low success rate in our study may be due to the longer follow-up period, difference in preoperative alignments, definition of success and various surgical procedures. In our study, unilateral RR was the only significant factor related to better surgical outcomes in small angle exotropia after multivariate analysis which is in line with our previous reports^27,28^. In contrast, Dadeya *et al*.^23^ found that ULR is an attractive procedure in the treatment of exotropia of 25 to 30 PD. After 3-years of follow-up, 78% of the patients obtained satisfactory results between 5 PD of esotropia and 5 PD of exotropia. In a prospective study of 20 children with exotropia ranging from 15 to 25 PD, ULR appeared to be equally effective as BLR in small to moderate angle exotropia, however, the follow-up period was only 3 months^25^. Kim *et al*.^24^ compared the long-term surgical outcomes between RR and ULR in 180 children with intermittent exotropia of 20 to 25 PD and found that after more than 2 years of follow-up, surgical success was achieved in 61% in the ULR group and 56% in the RR group. Conversely in our study, success rates were 34% after ULR and 58% after RR, showing superior results after unilateral RR in small angle exotropia patients of 14 to 20 PD. The number of patients who received BLR was too small for statistical comparison.

Several factors have been reported to affect outcomes after surgery such as age at surgery, preoperative angle of deviation, lateral incomitance, type of exotropia, amblyopia, anisometropia and sensory status^29–36^. In our study, good surgical outcomes were related to older age and initial good stereopsis, although this association was not significant after multivariate analysis. Regarding the optimal age for surgery in intermittent exotropia, it remains controversial between many authors. Jampolsky^31^ preferred delayed surgery until the age of 4 in order to avoid overcorrection in visually immature infants. Backer and Davies^32^ reported that patients who had surgery after the age of 4 showed better functional results. In contrast, Knapp^33^ advocated early surgery. In our study, the reason for good motor outcome in older children may be better fusional ability, stereopsis and accuracy of measuring the angle of exodeviation^37^. In terms of stereopsis, Lee *et al*.^10^ addressed the clinical course of small angle exodeviation and found that the initial stereoacuity was worse in patients whose angle of exodeviation progressed to 20 PD or greater.

Surgery holds risk of certain adverse events such as persistent overcorrection, infection and perforation. Persistent overcorrection may result in amblyopia and loss of stereopsis. In our study, 5 patients (6%) of the surgery group showed final overcorrection. Three of them had an angle of 10 PD or less and maintained fusion with prism glasses after RR. Two patients who each underwent RR and ULR procedure resulted in a large angle of consecutive esotropia of 30 and 26 PD at 4.5 and 7.1 years after surgery. Both of them were treated with botulinum toxin injection into the medial rectus muscle, and finally showed an esotropia of 10 PD and 14 PD maintaining fusion with prismatic correction^38,39^. In terms of recurrence, 45% required reoperation for recurrent exotropia after 3.3 ± 1.7 years, and the rate may even increase with time owing to the continuous tendency of exotropic drift^12^. In contrast to surgery, the risk of observation is much less devastating^9,40^. Only 10% of patients in the observation group showed a decrease in fusional control and stereopsis in our study, which was not significantly different from the surgery group. Considering the risk of surgical complications and the stable course of small angle exotropia, patients do not seem to need early surgery in most cases. If cosmetic problems are not significant, surgical intervention could be delayed without concern of permanent loss of binocular function in young patients with small angle intermittent exotropia, even in those with poor fusional control at distance.

There are several limitations in this study. First, because of its retrospective nature, treatment options and surgical indications were not randomized. To minimize selection bias in this study, the two groups were matched by their initial angle of deviation at distance. Second, the measurement of exodeviation and the diagnosis of intermittent exotropia were made by two examiners (J.M.H. and H.K.Y.). This may possibly affect the result, but relatively uniform examinations and surgical indications were followed in our hospital and every individual patient was examined by the same examiner at each follow-up examinations. Despite these limitations, to the best of our knowledge, this is the first comparative study reporting long-term outcomes of conservative management and surgical intervention for small angle exotropia. Future longitudinal prospective studies are mandatory to establish evidence-based guidelines for the management of small angle exotropia and optimal timing of surgery.

In conclusion, surgical intervention did not appear to be more beneficial for the treatment of small angle exotropia, compared to those who underwent conservative management. Approximately half of patients with small angle exotropia achieved good motor outcome after an average follow-up of 4 years in both groups. The type of surgery, namely unilateral RR procedure, was the only predictive factor associated with long-term good motor outcome after surgery.

## Methods

A retrospective review of medical records was performed on consecutive patients with intermittent exotropia who visited Seoul National University Bundang Hospital between 2006 and 2012. The inclusion criteria were patients who were aged 3 to 13 and diagnosed with small-angle intermittent exotropia of 14 to 20 PD. Patients with congenital anomalies, neurologic disorders, paralytic or restrictive strabismus, ocular disease other than strabismus and infantile exotropia were excluded. The patients were then divided into two groups; observation group and surgery group. Patients in the observation group required a minimum follow-up period of at least 2 years after the initial diagnosis, and patients in the surgery group required a minimum follow-up period of at least 2 years after operation. A total of 128 patients were enrolled in the observation group and 168 patients in the surgery group. Age and the angle of deviation at distance were randomly matched between the two groups; data from the initial examination of the observation group and preoperative examination of the surgery group were used for matching (Excel, Microsoft Corp., Redmond, WA). Finally, 82 patients in the surgery group and 82 age and angle of deviation- matched patients in the surgery group were identified. The study protocol was approved by the Institutional Review Board of Seoul National University Bundang Hospital and adhered to the tenets of the Declaration of Helsinki. The requirement for obtaining informed patient consent was waived due to the retrospective nature of the study.

### Ophthalmologic examination

The angle of deviation was measured by prism and alternate cover test with refractive correction at near (1/3 m) and distance (6 m). The presence of fixation preference and fusional control were determined with repeated examinations of the cover-uncover test. Fusional control of exodeviation was classified into three grades as good (manifest deviation only after cover testing and resumes fusion rapidly without need for a blink or refixation, fair (blinks or refixates to control the deviation after disruption with covering test) and poor (manifest deviation spontaneously occurs in the office without any form of fusion disruption)^41^. Improved fusional control was defined as an improvement of one grade or more at distance. Refractive errors were analyzed as spherical equivalent values which were obtained from cycloplegic refraction. Anisometropia was defined when a spherical equivalent difference between two eyes was more than 1.50 diopters (D). Amblyopia was defined when a difference between monocular best corrected visual acuities was 2 lines or more. Lateral incomitance was defined when a change in lateral gaze compared with the primary position was 5 PD or more. Randot stereoacuity test (Stereo Optical Company, Inc., Chicago, IL) was used to evaluate sensory status in cooperative patients. We defined good stereoacuity as 100 seconds of arc or better and improved stereopsis as a decrease of more than 2 octaves at final follow-up examination^42^.

### Surgical intervention

Surgery was recommended if there was one of the following reasons; (1) progressively increasing angle of deviation, (2) increasing frequency of deviation, (3) loss of binocular vision, or (4) cosmetic problems. The final decision was made by the patient/parents and surgeon. All of the patients were operated under general anesthesia by a single experienced surgeon (J.M.H) using the same surgical table. One of the following procedures was performed: (1) bilateral lateral rectus recession of 5 mm (BLR) or (2) Unilateral lateral rectus recession of 10 mm (ULR) or (3) unilateral MR resection based on the near deviation, with LR recession based on the distant deviation (RR). Surgery was based on the largest angle of deviation measured at both distance and near fixation.

### Main outcome measures

Primary outcome measures included the angle of deviation at near and distance, fusional control at near and distance, and near stereopsis at the final follow-up examination. In terms of estimating the final angle of exodeviation, 5 patients with consecutive esotropia were excluded from the surgery group as it may underestimate the average angle of exodeviation. Secondary outcome measures were factors associated with good or poor motor outcome. Good motor outcome (success) was defined as an exodeviation of less than 10 PD or esodeviation of less than 5 PD. Exodeviation of 10 PD or more and esodeviation of 5 PD or more were defined as poor outcome. Change in cycloplegic refractive errors from the initial to final visit was evaluated. Fast myopia progression^43^ was defined as an average myopic shift of −0.50 D or greater per year. Subgroup analysis was performed on patients with poor fusional control.

### Statistical analysis

Statistical analysis was performed using SPSS for Window version 22.0 (SPSS Inc, Chicago, Illinois, USA). The student’s t-test, chi-square test, Mann-Whitney U test and Fisher’s exact test were used to compare the patients’ characteristics and outcomes. Univariate analysis and multivariate logistic regression were performed to identify factors affecting outcomes including gender, age at onset, age at diagnosis/surgery, initial/preoperative angle of exodeviation at distance and near, refractive errors, anisometropia, amblyopia, dissociated vertical deviation, oblique dysfunction, lateral incomitance, vertical deviation, fixation preference, fusional control, stereopsis and type of surgery. P-values of <0.05 were considered to be statistically significant.

## Data Availability

Data supporting the findings of the current study are available from the corresponding author on reasonable request.
